# High Prevalence of Antinuclear Antibodies in Children with Thyroid Autoimmunity

**DOI:** 10.1155/2014/150239

**Published:** 2014-02-24

**Authors:** Maria Segni, Ida Pucarelli, Simona Truglia, Ilaria Turriziani, Chiara Serafinelli, Fabrizio Conti

**Affiliations:** ^1^Department of Pediatrics, Endocrinology Unit, “Sapienza” University of Rome, Via Regina Elena 324, 00161 Rome, Italy; ^2^Department of Internal Medicine and Medical Specialties, Rheumatology, “Sapienza” University of Rome, Rome, Italy

## Abstract

*Background*. Antinuclear antibodies (ANA) are a hallmark of many autoimmune diseases and can be detected many years before disease onset. Autoimmune thyroid diseases (AITD) are frequently associated with other organ- and non-organ-specific autoimmune disorders. *Objectives*. To assess the prevalence of ANA in pediatric patients with AITD and their clinical correlations. *Methods*. Ninety-three consecutive pediatric patients with AITD were enrolled (86 children with chronic lymphocytic thyroiditis and 7 with Graves' disease). ANA, anti-double DNA (anti-dsDNA) antibodies, anti-extractable nuclear antigen (anti-ENA), anti-cyclic citrullinated peptide antibodies (anti-CCP), and rheumatoid factor (RF) was obtained. Signs and symptoms potentially related to rheumatic diseases in children were investigated by a questionnaire. *Results*. ANA positivity was found in 66/93 children (71%), anti-ENA in 4/93 (4.3%), anti-dsDNA in 1/93 (1.1%), RF in 3/93 (3.2%), and anti-CCP in none. No significant differences were found between the ANA-positive and ANA-negative groups with respect to age, sex, L-thyroxine treatment, or prevalence of other autoimmune diseases. Overall, parental autoimmunity was found in 23%. *Conclusions*. ANA positivity was demonstrated in 71% of children with AITD. ANA positivity was not related to overt immune-rheumatic diseases. However, because the positivity of ANA can occur even many years before the onset of systemic autoimmune diseases, prospective studies are warranted.

## 1. Introduction

Antinuclear antibodies (ANA) are a marker of several autoimmune diseases including autoimmune thyroid disease (AITD) and Systemic Lupus Erythematosus (SLE) and they can be detected many years before disease onset [1–5]. ANA positivity can also be found in patients with malignant or infectious diseases as well as in healthy subjects [2–5].

The AITD, Graves' disease (GD), and chronic lymphocytic thyroiditis (CLT) are organ-specific autoimmune disorders that are defined by lymphocytic infiltration of the thyroid [6] and autoantibodies against thyroid antigens [i.e., thyroid peroxidase antibody (TPOAb), thyroglobulin antibody (TgAb), and anti-TSH-receptor antibody (TRAb)] [7]. AITD is frequently associated with other organ and non-organ-specific autoimmune disorders [8–10]. This association has been also reported in juvenile forms of chronic arthritis and SLE [11, 12]. ANA prevalence up to 45% has been reported in adult patients with AITD [13, 14]. To the best of our knowledge, so far, only one study analyzed ANA positivity in children with AITD [15]. We investigated the prevalence of serum ANA in pediatric patients with AITD and their association with signs and symptoms related to immune-rheumatic diseases.

## 2. Patients and Methods

We studied 93 consecutive children (86 patients with CLT and 7 with GD, 76 females) referred for AITD to the Pediatric Endocrinology Unit of “Sapienza” University in Rome. CLT was defined on the basis of the presence of thyroid autoantibodies more than two times the upper normal value (nv) ([TPOAb nv <20 IU/mL] and/or TgAb [nv <50 IU/mL]) and thyroid ultrasound evaluation showing reduced echogenicity compatible with thyroiditis, regardless of thyroid function. GD was defined by clinical and biochemical hyperthyroidism and by positivity for TRAb.

Written informed consent was obtained from parents. This study was approved by the ethical committee of our institution.

The enrolled patients underwent a complete physical examination and the clinical and laboratory data were collected in a standardized form, which includes demographics, past medical history with date of diagnosis, comorbidities, and previous and concomitant treatments. All children and parents were interviewed according to a questionnaire seeking signs and symptoms related to rheumatic diseases in children. The questionnaire took into account the following signs and symptoms: joint pain, joint swelling, back pain, morning stiffness, asthenia, Raynaud's phenomenon, xerostomia, xerophthalmia, pleuritis, and pericarditis. Children with signs and/or symptoms suggestive of immune-rheumatic diseases were examined by a rheumatologist (FC).

Patients underwent peripheral blood sample collection and sera were stored at −20°C until being assayed.

Free T3, free T4, TSH, TPOAb, and TgAb serum levels were determined in all patients and TRAb were determined in presence of hyperthyroidism. FT3 and FT4 were determined by RIA and TSH (upper normal value 3.5 *μ*U/mL) was determined by immunoradiometric assay (all by Byk Sangtec Diagnostica, Dietzbach, Germany). TRAb were detected by radioreceptor assay (Radim, Angleur, Belgium) and TPOAb and TgAb by immunoradiometric assay (ICN Pharmaceutical Inc., Costa Mesa, CA).

ANA were detected by indirect immunofluorescence assay (IIFA) on HEp-2 cells (ANA Nova LIte TM HEp-2, INOVA Diagnostics Inc., San Diego, CA 92131, USA). Anti-double DNA antibodies (anti-dsDNA) were investigated by *Crithidia luciliae *immunofluorescence test (CLIFT) (A. Menarini Diagnostics, 50131 Florence, Italy). ANA were considered positive at a titer ≥ 1 : 80, anti-dsDNA at a titer ≥ 1 : 10. Immunofluorscence intensity ranged from + to ++++. Serum antibodies against extractable nuclear antigen (anti-ENA screening and in the case of ENA positivity: Sm, RNP, SS-A, SS-B, Scl-70, Jo-1) were determined by ELISA (QUANTA Lite ENA 6, INOVA Diagnostics Inc., San Diego, CA 92131, USA). In addition, anti-cyclic citrullinated peptide antibodies (anti-CCP) and rheumatoid factor (RF) were determined. Anti-CCP were determined by fluoroenzyme-immunoassay (EliACCP), using immunoCAP 100 analyzer (Phadia AB, 75002 Uppsala, Sweden) and RF was determined by nephelometry using BN2 automate (N Latex RF kit, Siemens Healthcare Diagnostics Products, Marburg 35037, Germany).

## 3. Statistics

Statistical calculations were performed using SPSS for Windows Version 17 (SPSS, Chicago, IL, USA). Data were expressed as mean and standard deviation for continuous data. Chi-square or Fisher's exact test was carried out when appropriate*. P* values < 0.05 were considered statistically significant.

## 4. Results

Clinical and laboratory features of the enrolled patients are reported in Table 1(a). ANA positivity was found in 66/93 children (71%), anti-ENA was found in 4/93 (4.3%), anti-dsDNA antibodies were found in 1/93 (1.1%), RF was detectable in 3/93 (3.2%), and anti-CCP were found in none. ENA-specific autoantibodies were determined in 3 out of 4 anti-ENA-positive patients, 1 had anti-RNP positive, 1 had anti-Jo-1, and 1 was negative for specific autoantibodies. The ANA pattern was homogeneous in 61/66 (92.4%), coarse/fine speckled in 4/66 (6%), and nucleolar in 2/66 (3%). The IIFA intensity was +++ in 23/66 (34.8%), ++ in 24/66 (36.3%), and + in 19/66 (28.7%) cases.

Among the 93 children with AITD, 20 (21%) had at least one other autoimmune disease: 9 with celiac disease (CD), 2 with autoimmune gastritis (AG), 4 with type 1 diabetes mellitus (type 1 DM), 2 with alopecia, 2 with vitiligo, and 1 with autoimmune thrombocytopenia. We found that 3/7 (43%) of children with GD presented with one other autoimmune disease (1 CD, 1 type DM, and 1 AG), versus 17/86 (20%) with CLT (8 CD, 1 AG, 3 type 1 DM, 2 alopecia, 2 vitiligo, and 1 autoimmune thrombocytopenia). The difference in the frequency of autoimmune disease associated with CLT versus GD was not significant.

Clinical and laboratory features of ANA-positive and ANA-negative patients are compared in Table 1(b). No significant differences were found between the ANA-positive and ANA-negative groups with respect to age, sex, L-thyroxine treatment, TSH, TPOAb, and TgAb levels, or presence of other autoimmune diseases in them and in their parents. Associated autoimmune diseases in ANA-positive and ANA-negative children and their parents are reported in Table 2. Hereby ANA-positive children did not differ for the frequency of additional autoimmune disease, but there was a higher percentage of parental autoimmunity in ANA-negative patients, however, without significance.

Signs and symptoms as investigated by questionnaire are detailed in Table 3. No significant differences were found between ANA-positive and ANA-negative children with AITD.

Children with persistent joint pain were referred to a rheumatologist (FC). Children with Raynaud's phenomenon underwent capillaroscopy. No evidence of SLE, RA, or other systemic autoimmune diseases was found.

## 5. Discussion

AITD is a common autoimmune disease and it is frequently associated with other organ and non-organ-specific autoimmune disorders [8–10]. A variable ANA prevalence up to 45% has been reported in AITD adult patients [13, 14].

ANA can also be detected in different autoimmune disorders (i.e., SLE, Sjogren's syndrome, progressive systemic sclerosis, mixed connective-tissue disease, juvenile idiopathic arthritis, primary autoimmune cholangitis, and autoimmune hepatitis) as well as in infections (2,4). In particular, ANA can be detected in over ninety percent of patients with SLE, a multifactorial autoimmune disease, involving genetic and environmental factors, characterized by a wide range of autoantibodies and clinical manifestations [4, 16–25].

ANA can be also found in healthy people [2]. A recent cross-sectional analysis of 4754 individuals older than 12 years showed a prevalence of ANA of 13.8% [26]. A similar prevalence of 12.6% was reported in healthy children, with higher titers found between 5 and 10 years of age [27]. To our knowledge, only one previous study investigated ANA prevalence in children with AITD. The authors, using a cut-off of 1 : 40 for ANA IIFA on HEp-2 cells, demonstrated an incidence of ANA positivity significantly higher in patients with untreated GD (71%) than in CLT (33%) [15]. In addition, ANA positivity was identified as a predictive factor for poor response to antithyroid drugs in GD. In our study, CLT represented 92% of total enrolled patients versus 36% in the previous study. Since we analyzed GD regardless of treatment and we included only 7 children affected with GD, we were not able to correlate GD activity with ANA positivity. In CLT patients, we did not find any difference in L-thyroxine treatment between ANA-positive and ANA-negative children. In contrast, a correlation between increased ANA levels and reduced thyroid volumes was reported in adult patients affected by vitiligo and AITD [28].

To detect serum ANA, we used the same method of Inamo and Harada [15], IIFA on HEp-2 cells, that is considered the most reliable method to search ANA [2, 29]. Using a higher cut-off value of 1 : 80, we found detectable ANA in 71% of children with AITD and the IIFA homogeneous pattern was detected in 92% of cases. Titers and pattern of ANA have been proposed to be a critical parameter for discriminating ANA, a homogeneous pattern being suggestive of rheumatic diseases [30]. A nuclear fine speckled pattern was reported in ANA-positive healthy children in 77% of cases [27].

We did not find any significant difference in ANA-positive and ANA-negative group with respect to age, sex, LT4 treatment, TSH, TPOAb and TgAb levels, and prevalence of other autoimmune diseases in the children or in the parents. No differences were detected investigating signs and symptoms of systemic diseases between ANA-positive and ANA-negative group. Altogether, joint pain was referred by 19% of patients, asthenia was referred by 19%, and Raynaud's phenomenon was present in 4.3%, as well as xerostomia and xerophtalmia. Because of the fact that hypothyroidism itself can cause symptoms as asthenia, we confirmed symptoms when children were euthyroid. Torok and Arkachaisri [31] in children and adolescents referred to a rheumatological center for ANA positivity and investigated for AITD, found arthralgias and fatigue more frequently in ANA-positive/antithyroid antibodies-negative subjects. The prevalence of positive antithyroid antibodies in this study was 30% versus 3-4% in general pediatric population [32], and hypothyroidism was found in 14%. It may suggest that to screen for AITD may be worthwhile in apparently healthy children with ANA positivity.

The clinical and biological meaning of ANA is still debated [29, 33, 34]. It has been speculated that a defect in the mechanisms involved in the engulfment of dead cell with inappropriate clearance of self-nucleic acids can cause autoimmune diseases. A deficiency of clearance of apoptotic cells is considered one of the causes of SLE and may underlie autoimmunity itself [35–37]. Activation of the innate and acquired immune response, which can be induced by infection, inflammation, or tissue injury, may impact on the development of autoimmunity in the thyroid [38, 39]. We can speculate that such a high prevalence of ANA in juvenile AITD may be read as a link to the involvement of self nucleic acids in the development of AITD.

An important point of our study is the observation of parental autoimmunity in up to 30% with only slightly lower percentage (20%) in ANA-positive children. Whether this reflects (epi-)genetic heritability or a shared familial environment triggering autoimmunity in parents and children needs to be investigated in prospective family studies.

In conclusion, although we analyzed a limited number of cases, our study demonstrated ANA positivity in over 70% of children with AITD. ANA positivity at the time of the study was not related to overt immune-rheumatic diseases.

In our opinion, the finding of ANA positivity in children and adolescents affected by AITD needs a careful reevaluation: it can be interpreted as a manifestation of “activated autoimmunity” without clinical relevance at the time of the study. However, because the positivity of ANA and other non-organ-specific autoantibodies can occur even many years before the onset of systemic autoimmune diseases [5, 40, 41], we think that prospective studies are warranted, especially in subjects positive for anti-dsDNA and anti-ENA.

## Figures and Tables

**Table:** (a)

Characteristic	Patients (N = 93)
F/M	76/17
CLT (*N*)	86
GD (*N*)	7
Age at diagnosis (mean years ± SD)	10.2 ± 3.9
Age at sampling (mean years ± SD)	12.1 ± 4.86
Duration of disease (mean years ± SD)	2.79 ± 3.67
CLT on LT4 treatment (*N*/%)	47/86 (54.6%)
TSH (µIU/mL ± SD)	2.8 ± 1.8
TPOAb (IU/mL ± SD)	819 ± 908
TgAb (IU/mL ± SD)	512 ± 605
Associated autoimmune disease in children (*N*/%)	20/93 (21%)
Associated autoimmune disease in CLT (*N*/%)	17/86 (20%)
Associated autoimmune disease in GD (*N*/%)	3/7 (43%)
Autoimmune disease in parents (*N*/%)	43/186 (23%)

**Table:** (b)

Patients	ANA positive (*N* = 66)	ANA negative (*N* = 27)	*P*
F/M	56/10	20/7	

CLT (*N*/%)	62 (72%)	24 (28%)	0.7
GD (*N*%)	4 (57%)	3 (43%)	0.7
Age at diagnosis (mean years ± SD)	10.4 ± 3.8	9.9 ± 4.2	0.61
Age at sampling (mean years ± SD)	12.4 ± 4.8	11.4 ± 4.4	0.32
TSH (µIU/mL ± SD)	3.01 ± 1.77	2.48 ± 1.86	0.2
TPOAb (IU/mL ± SD)	903 ± 941	614 ± 803	0.61
TgAb (IU/mL ± SD)	519 ± 593	494 ± 666	0.85
LT4 therapy (CLT) (*N*/%)	31/62 (50%)	16/27 (66%)	0.99

F/M: females/males; CLT: chronic lymphocytic thyroiditis; GD: graves' disease; TSH: thyroid stimulating hormone; TPOAb: thyroid peroxidase antibodies; TgAb: thyroglobulin antibodies; LT4: L-thyroxine.

**Table 2:** Associated autoimmune diseases in ANA-positive and ANA-negative children with AITD and in their parents.

Patients	ANA positive (*N* = 66)	ANA negative (*N* = 27)	*P*
Associated autoimmune diseases (*N*/%)	14/66 (21%)	6/27 (22%)	0.87
Celiac disease (*N*)	6	3	
Type 1 DM (*N*)	2	2	
Autoimmune gastritis (*N*)	2	0	
Vitiligo (*N*)	2	0	
Alopecia (*N*)	1	1	
Autoimmune thrombocytopenia (*N*)	1	0	

Parents	66 mothers, 66 fathers	27 mothers, 27 fathers	

Autoimmune diseases in parents (*N*/%)	27/132 (20.4%)	16/54 (29.6%)	0.24

Autoimmune diseases in mothers (*N*)	18	10	
AITD (*N*)	14	10	
SLE (*N*)	1	0	
Sjogren's syndrome (*N*)	1	0	
Autoimmune thrombocytopenia (N)	1	0	
Autoimmune diseases in fathers (*N*)	9	6	
AITD (*N*)	8	4	
Vitiligo (*N*)	1	1	
Rheumatoid arthritis (*N*)	0	1	

Type 1 DM: type 1 diabetes mellitus; AITD: autoimmune thyroid disease; SLE: systemic lupus erythematosus.

**Table 3:** Positive answers at questionnaire in ANA-positive and ANA-negative children with AITD.

Question	ANA positive (*N* = 66)	ANA negative (*N* = 27)	*P*
Joint pain	12 (18%)	5 (18%)	0.87

Joint swelling	0 (0%)	0 (0%)	—
Morning stiffness	1 (1.5%)	0 (0%)	—
Back pain	1 (1.5%)	1 (3.7%)	—
Asthenia	14 (21%)	4 (14%)	0.47
Raynaud's	3 (4.5%)	1 (3.7%)	0.63
Xerostomia	3 (4.5%)	1 (3.7%)	0.63
Xerophthalmia	3 (4.5%)	1 (3.7%)	0.63
Pleuritis	0	0	—
Pericarditis	0	0	—
